# Effects of longitudinal changes in Charlson comorbidity on prognostic survival model performance among newly diagnosed patients with hypertension

**DOI:** 10.1186/s12913-016-1910-8

**Published:** 2016-11-22

**Authors:** Peter Rymkiewicz, Pietro Ravani, Brenda R. Hemmelgarn, Finlay A. McAlister, Danielle A. Southern, Robin Walker, Guanmin Chen, Hude Quan

**Affiliations:** 1 Measurement and Evaluation, Mosaic Primary Care Network, Calgary, AB Canada; 2 Department of Community Health Sciences, Cumming School of Medicine, University of Calgary, Calgary, AB Canada; 3 O’Brien Institute for Public Health, Cumming School of Medicine, University of Calgary, Calgary, AB Canada; 4 Faculty of Medicine and Dentistry, University of Alberta, Edmonton, AB Canada; 5 Libin Cardiovascular Institute of Alberta, Cumming School of Medicine, University of Calgary, Calgary, AB Canada; 6 Alberta Health Services, Calgary, AB Canada; 7 University of Calgary, 3E23 3rd fl TRW, 3280 Hospital Drive NW, Calgary, T2N 4Z6 AB Canada

**Keywords:** Charlson, Comorbidity, Model performance, Hypertension, Longitudinal change

## Abstract

**Background:**

To assess the use of updated comorbidity information over time on ability to predict mortality among adults with newly diagnosed hypertension.

**Methods:**

We studied adults 18 years and older with an incident diagnosis of hypertension from Alberta, Canada. We compared the prognostic performance of Cox regression models using Charlson comorbidities as time-invariant covariates at baseline (TIC) versus models including Charlson comorbidities as time-varying covariates (TVC) using Akaike Information Criterion (AIC) for testing goodness of fit.

**Results:**

The strength of the association between important prognostic clinical variables and mortality varied by modeling technique; for example, myocardial infarction was less strongly associated with mortality in the TIC model (Hazard Ratio 1.07; 95% Confidence Interval (CI): 1.05 to 1.1) than in the TVC model (HR 1.20; 95% CI: 1.18 to 1.22). All TVC models slightly outperformed TIC models, regardless of the method used to adjust for comorbid conditions (individual Charlson Comorbidities, count of comorbidities or indices). The TVC model including all 17 Charlson comorbidities as individual independent variables showed the best fit and performance.

**Conclusion:**

Accounting for changes in patient comorbidity status over time more accurately captures a patient’s health risk and slightly improves predictive model fit and performance than traditional methods using TIC assessment.

## Background

Comorbidity is an important component of individual risk and health status. It has been shown to be an important determinant of health care utilization, predictor of health outcomes and mortality. Studies using large administrative data-based cohorts to predict death have historically adjusted for individual risk on the basis of comorbidities present at baseline [1, 2]. However, disease severity, and the presence and progression of comorbidities, is not static. Risk adjustment for comorbidities often ignores the nature of disease progress in the observational period. Little is known about how comorbidities developing during follow-up and/or how changes in disease severity over time impact the ability to predict important outcomes such as mortality.

The Charlson comorbidity index (CCI) [3] was developed as a prognostic classification and weighting methodology that predicts mortality based on disease burden. Short-term studies predicting 30-day and 1-year mortality using time-invariant baseline Charlson comorbidities adjustment have shown good performance. Recent studies suggest that predictive models solely relying on baseline measures of comorbidity may be less accurate in longer follow-up studies where novel conditions or changes in disease severity are more likely to occur [2, 4–6]. However, few studies account for changes in disease state after baseline and there is contradictory evidence about the value of time-varying effects in prognostic model performance [1, 7].

The purpose of this study was to determine whether predictive models accounting for changes in patient comorbidity status over time may more accurately capture a patient’s health risk and better predict long-term survival than models using only baseline measures of comorbidity.

## Methods

### Data sources

Administrative databases used in the study included: (1) Hospital discharge abstracts (DAD), (2) the Alberta Health Care Insurance Plan (AHCIP) registry, (3) Physician billing claims, and (4) vital statistics. Clinical visit information for all patients discharged from Alberta hospitals is abstracted and recorded. Clinical information in DAD includes up to 16 possible diagnoses abstracted using International Classification of Disease (ICD), 9^th^ Revision (i.e., ICD-9), ICD-9 Clinical Modification (ICD-9-CM) and up to 25 possible diagnoses abstracted using ICD-10 Canadian Modification (i.e., ICD-10-CA) [8]. The AHCIP includes individually identifiable demographic information (Personal Health Number (PHN), date of birth, sex, and postal code) for all Alberta residents eligible to receive universally covered healthcare services. Physician fee-for-service and shadow billing claims submitted to the provincial government includes information on type of provider, patient and clinic information, with at least one, but up to three, ICD-9 diagnostic codes for both outpatient as well as inpatient hospital services provided by Alberta physicians to eligible Albertans who have registered under the AHCIP.

### Study population

The study population included all adults aged 18 years and older who were residents of the Province of Alberta and newly diagnosed with hypertension. We identified individuals with hypertension, using relevant ICD-9 (401.x, 402.x, 403.x, 404.x, or 405.x) and ICD-10-CA codes (I10.x, I11.x, I12.x, I13.x, or I15.x), and assigned the earliest date of a physician visit or hospital separation as the index date using methods previously described [9]. We excluded patients with an index date between April 1, 1994 and March 31, 1997 (the washout period), thereby focusing on patients newly diagnosed with hypertension between April 1, 1997 and March 31, 2009. We excluded patients from the study if they met any of the following criteria: 1) hypertensive patients diagnosed in the washout period (between April 1, 1994 and March 31, 1997); 2) patients with myocardial infarction, heart failure or stroke during the 3 years leading up to the study or within 30 days following the diagnosis of hypertension; 3) patients who died the same day that they were diagnosed with hypertension; 4) patients with less than 1-year of follow.

### Outcome and independent variables

The outcome variable was all-cause mortality between April 1, 1997 and March 31, 2009. We followed patients from diagnosis until death or the end of the study (March 31, 2010). We measured survival time from the date of the initial hypertension diagnosis to the date of death or the last observation date and censored observations at the AHICP coverage end date or study end date of March 31, 2010 if death did not occur. Deaths were identified from Vital Statistics [10].

The primary independent variables were the 17 Charlson comorbidities present at baseline (included in both time-invariant covariate (TIC) and in time-varying covariate (TVC) analyses) or developing between April 1 1997 and March 31 2009 (included only in TVC analysis). We obtained comorbidity data from the physician claims and DAD databases and age and sex information from the APCIP. We assigned median household income quintiles (lowest quintile Q1 to highest quintile Q5), a proxy for socioeconomic status, and rural or urban residential location based on their residential postal code, mapped to 2006 Statistics Canada Census data [11].

The onset of a patient’s disease comorbidity was determined as the earliest date of diagnosis obtained from DAD or physician claims data following a patient’s index of hypertension diagnosis date. If more than one record was identified for the same condition, we chose the first date of encounter as date of the chronic condition. In TVC models, we used the last observation carried forward approach. Once identified, each condition was considered ongoing, with models capturing onset of comorbidity in their original form along with time at risk in the period between diagnosis and the remainder of the study [1].

### Statistical analysis

We used descriptive statistics to summarize data on age, sex, socio-economic status and area of residence. We analyzed survival data using the Kaplan-Meier method and Cox Regression, and tested associations using the log-rank test and Wald test with two-sided *P* < 0.05 as statistical significance level. We calculated crude hazard ratios (cHR) and adjusted hazard ratios (aHR) using non-disease reference populations for Cox’s regression models and reported 95% Confidence Intervals (95% CI) as a measure of uncertainty around each estimate.

We used four methods to adjust for patient case-mix for both TIC and TVC models along with other potential confounders including age, sex, median household income, and area of residence. These four methods included 1) 17 individual Charlson comorbidities, 2) the original Charlson comorbidity index summary score, 3) an updated Charlson comorbidity index summary score using 12 of the original 17 comorbidities and 4) a count of Charlson comorbidities [12].

We studied the slope of the line of the Schoenfeld residuals over time to verify that the hazard proportionality assumption was met. Because of the large sample size, we excluded violations if the coefficient of the slope was within the pre-specified range of 0 ± 0.05, regardless of whether it was statistically significantly different from zero. In sensitivity analyses we checked if the log-hazard ratios differed by more than 10% when time was split in quarterly segments.

We fit eight multivariate prognostic survival models controlling for a standard set of confounding variables; one set of TIC and one set of TVC Models using standard Cox regression including each of all four methods described above. Each Cox regression included one condition such as myocardial infarction (presence or absence). We compared model fit using the Akaike Information Criterion (AIC), which does not require the models under comparison to be nested. Generally, improved model performance (better fit and parsimony) is supported by a decrease in the AIC score [13].

## Results

Between April 1 1997 and March 31 2009, 456,263 newly diagnosed Alberta hypertensive patients were identified. After excluding those who had died on date of diagnosis (*n* = 2529), there were 453,734 patients. Patients predominantly lived in urban areas (80.7%) and 50.6% were male. The median age was 57.5 and 67.9% were younger than 65 years. There were 29,717 (6.5%) patients missing median income information and 1255 (.27%) patients with missing residence information. Missing data on these variables were included in the analysis. During the follow-up (a median follow-up time of 5.75 years, inter-quartile range (IQR) 5.74 to 5.76 years) 72,490 died and mortality was 15.98% (95% CI: 15.86 to 16.01) (Table 1).

**Table 1:** Study population characteristics at baseline

Study population characteristics	Number	%
Total Patients	453,734	
Age, years		
20-49	139,110	30.6
50-64	168,998	37.2
65-74	81,930	18.1
≥75	63,696	14
Mean (SD)	57.52 (14.63)	
Median Income quintile		
1 (Lowest)	91,891	20.3
2	86,694	19.1
3	84,876	18.7
4	81,269	17.9
5 (Highest)	79,287	17.5
Missing	29,717	6.5
Region of Residence		
Urban	366,226	80.7
Rural	86,253	19
Missing	1255	0.3
Mortality during follow-up	72,490	15.98
Duration of Follow-up		
Median Years (IQR)	5.75 (5.74-5.76)	

*Abbreviations*: *SD* standard deviation, *CI* confidence interval, *IQR* inter-quartile range

The prevalence of the 17 Charlson Comorbidities differed substantially between baseline and the end of the study period (Table 2), increasing from 36.8% of patients with at least one comorbid condition to 60.4% at the end of study. The prevalence for each comorbidity increased substantially during the study period, from 1.74 times (chronic obstructive pulmonary disease) to 3.81 times (metastatic solid tumors).

**Table 2:** Prevalence of 17 individual Charlson comorbidities at baseline and at the end of the study

	Baseline		At End of Study	
	*n*	%	*n*	%
Charlson Comorbidities				
Myocardial infarction	27,304	(6.02)	53,774	(11.85)
Congestive heart failure	26,041	(5.74)	60,426	(13.32)
Peripheral vascular disease	13,399	(2.95)	33,863	(7.46)
Cerebrovascular disease	23,881	(5.26)	56,297	(12.41)
Hemiplegia or paraplegia	3738	(0.82)	8536	(1.88)
Dementia	8890	(1.96)	31,659	(6.98)
Chronic pulmonary disease	70,227	(15.48)	122,785	(27.06)
Rheumatologic disease	8161	(1.80)	18,223	(4.02)
Peptic ulcer disease	11,674	(2.57)	27,142	(5.98)
Diabetes without chronic complications	31,098	(6.85)	58,389	(12.87)
Diabetes with chronic complications	7503	(1.65)	23,390	(5.16)
Renal disease	10,231	(2.25)	34,560	(7.62)
Any malignancy, including leukemia and lymphoma	26,853	(5.92)	69,637	(15.35)
Metastatic solid tumor	4784	(1.05)	18,231	(4.02)
Mild liver disease	6524	(1.44)	16,860	(3.72)
Moderate or severe liver disease	968	(0.21)	2782	(0.61)
AIDS/HIV	230	(0.05)	407	(0.09)
Number of Charlson Comorbidities				
0	285,417	(63.2)	179,802	(39.6)
1-2	140,101	(28.1)	181,353	(40.0)
≥3	28,216	(8.7)	92,579	(20.4)

Because mortality was relatively low in this cohort, there was no death in some subgroups. In fact, while the crude analysis showed that patient survival was affected by all 21 independent variables, we calculated survival time for 10 comorbidity-defined subgroups. Conditions not reaching a reportable endpoint had median survival longer than the 12-year study follow-up. Comorbidities associated with the shortest median survival time included metastatic solid tumors (*n* = 4784, 2.17 years, 95% CI 1.97-2.39), dementia (*n* = 8890, 3.67 years, 95% CI 3.58-3.79) and moderate or severe liver disease (*n* = 968, 4.10 years, 95% CI 3.72-5.15) years. Comorbidities associated with increased mortality included hemiplegia and paraplegia (cHR: 7.90, 95 % CI: 7.51-8.43), renal disease (cHR: 8.67, 95% CI 8.34-9.08), cerebrovascular disease (cHR: 9.84, 95 % CI: 9.58-10.14), peripheral vascular disease (cHR: 9.16, 95 % CI: 8.80-9.44) and malignancy (cHR: 10.38, 95 % CI: 10.00-10.73).

While the direction of the association between predictor and mortality remained the same for all variables included in the TIC and TVC models, the strength of each association (and even whether it was statistically significant or not) differed between the two model formulations for several important prognostic variables (Tables 3 and 4). aHR estimates for seven comorbidities increased in the TVC model compared with that in TIC models: myocardial infarction, congestive heart failure, cerebrovascular disease, hemiplegia/paraplegia, mild or sever liver disease, cancer, metastatic solid tumors. For example, the aHR (95% CI) for myocardial infarction was 1.07 (1.05-1.1) in the TIC model and 1.2 (1.18-1.22) in the TVC model and for chronic obstructive pulmonary disease was 1.18 (1.13-1.24) in the TIC model and 1.03 (0.99- 1.07) in the TVC model.

**Table 3:** Comparison of crude and adjusted hazard ratios (95 % confidence interval) between the baseline and time-varying covariate (TVC) models for all 17 individual Charlson comorbidities

	Baseline		TVC	
	cHR^a^ (95 % CI)	aHR^b^ ((95 % CI)	cHR^a^ ((95 % CI)	aHR^b^ ((95 % CI)
Sex (Male)	1.15 (1.14-1.17	1.23 (1.22-1.26)	1.15 (1.14-1.17)	1.15 (1.14-1.17)
Age	1.05 (1.05-1.06)	1.04 (1.04-1.04)	1.06 (1.05-1.06)	1.03 (1.03-1.03)
Region of Residence				
Urban	1.01 (.99-1.03)	1.10 (1.08-1.12)	1.01 (.99-1.03)	1.09 (1.07-1.11)
Income Quintile				
(Compared to 1- Lowest)				
2	.88 (.86-.90)	.95 (.93-.98)	.88 (.86-.90)	.97 (.95-.99)
3	.81 (.79-.83)	.92 (.90-.94)	.81 (.79-.83)	.94 (.92-96)
4	.69 (.67-.71)	.86 (.85-.89)	.69 (.67-.71)	.91 (.88-.95)
5 – Highest Income Quintile	.65 (.63-.66)	.85 (.82-.87)	.65 (.63-.66)	.93 (.90-.95)
Charlson Comorbidities				
Myocardial infarction	2.2 (2.15-2.25)	1.07 (1.05-1.1)	2.89 (2.84-2.94)	1.2 (1.18-1.22)
Congestive heart failure	4.19 (4.11-4.27)	1.77 (1.73-1.81)	5.21 (5.13-5.29)	1.88 (1.84-1.92)
Peripheral vascular disease	2.99 (2.9-3.07)	1.24 (1.2-1.28)	3.31 (3.24-3.38)	1.16 (1.14-1.19)
Cerebrovascular disease	2.87 (2.8-2.93)	1.28 (1.24-1.31)	3.65 (3.59-3.72)	1.41 (1.38-1.43)
Hemiplegia or paraplegia	7.64 (7.43-7.85)	2.36 (2.29-2.43)	8.82 (8.66-8.98)	2.89 (2.83-2.95)
Dementia	1.88 (1.85-1.91)	1.31 (1.29-1.34)	2.16 (2.12-2.19)	1.3 (1.28-1.32)
Chronic pulmonary disease	1.69 (1.61-1.76)	1.18 (1.13-1.24)	1.62 (1.56-1.67)	1.03 (0.99-1.07)
Rheumatologic disease	1.67 (1.61-1.73)	1.1 (1.06-1.14)	2.02 (1.96-2.07)	1.1 (1.08-1.13)
Peptic ulcer disease	2.31 (2.21-2.42)	1.69 (1.6-1.78)	2.66 (2.58-2.74)	1.56 (1.51-1.62)
Diabetes without chronic complications	2.18 (2.13-2.23)	1.3 (1.27-1.33)	2.16 (2.12-2.2)	1.1 (1.07-1.12)
Diabetes with chronic complications	3.27 (3.14-3.4)	1.41 (1.35-1.48)	3.8 (3.71-3.9)	1.27 (1.23-1.31)
Renal disease	3.45 (3.28-3.62)	1.44 (1.36-1.52)	4.18 (4.04-4.33)	1.36 (1.31-1.41)
Any malignancy, including leukemia and lymphoma	3.52 (3.41-3.64)	1.63 (1.57-1.69)	4.78 (4.68-4.87)	1.72 (1.69-1.76)
Metastatic solid tumor	3.11 (3.04-3.18)	1.5 (1.47-1.54)	4.49 (4.42-4.57)	1.87 (1.83-1.90)
Mild liver disease	5.7 (5.23-6.21)	1.97 (1.79-2.17)	7.1 (6.73-7.49)	2.1 (1.98-2.23)
Moderate or severe liver disease	8.34 (8.04-8.65)	3.58 (3.43-3.73)	13.26 (12.99-13.53)	5.15 (5.03-5.28)
AIDS/HIV	1.86 (1.42-2.44)	1.72 (1.28-2.31)	2 (1.62-2.46)	1.65 (1.32-2.06)

^a^ *cHR* crude hazard ratio *AIC* Akaike Information Criterion

^b^ *aHR* adjusted hazard ratio-adjusted model included sex age area of residence and median household income

**Table 4:** Comparison of crude and adjusted hazard ratios (95 % confidence interval) between the baseline and time-varying covariate (TVC) models for all 17 individual Charlson comorbidities

Modeling Methods	Baseline AIC^a^	TVC AIC
1. 17 Individual comorbidities (17 variables)	1,720,126	1,670,491
2. Original Charlson Weighting Method (1 variable)	1,723,116	1,679,069
3. Updated Charlson Weighting Method (1 variable)	1,724,610	1,677,164
4. Count of up to 17 comorbidities (1 variable)	1,724,769	1,691,347

^*a*^ *cHR* crude hazard ratio *AIC* Akaike Information Criterion

Fit and performance analyses shows that models including all 17 Charlson comorbidities as individual covariates out-performed models using summary measures. These results were consistent in nested model comparisons for both TIV and TVC models. Based on these findings, we compared the performance of these models when all 17 Charlson comorbidities were included as individual covariates. The TVC model out-performed the baseline model achieving the lowest AIC = 1,670,491 (Tables 3 and 4).

## Discussion

We used a large cohort of adults with newly diagnosed hypertension to compare the performance of survival models according to the use of comorbidity data either at baseline (TIC approach) or with information updated during follow-up (TVC approach)). In this cohort of 475,345 newly diagnosed hypertensive patients identified over a 12 year period in Alberta, Canada, and followed for an average of  6 years, we found that prognostic models using updated disease occurrence information were more accurate than the baseline method in predicting individual risk of death.

In our study of relatively healthy adults with a new diagnosis of hypertension, the comorbidity burden increased significantly over time; nearly one quarter of patients developed at least one new comorbid condition following their initial diagnosis of hypertension. Consistent with other studies, the prevalence of Charlson comorbidities differed substantially between baseline and the follow-up end [1].

In order to capture mortality in people newly diagnosed with hypertension, long-term follow-up is necessary. Our median patient follow-up time was 5.75 years while our projected median survival time was in excess of 20-years. As a result, deaths were infrequent. Supported by previous study findings [14], we observed that TVC models had better fit and were more accurate in predicting mortality than TIC models. While differences in a healthy population were considered small, capturing change in comorbidities throughout the study period would improve prediction of individual risk. This may have implications for patient management and may alter the course of patient care to include aggressive prevention strategies as well as risk estimation for future studies.

TVC models captured incremental changes in disease state over time based on the serial measurement method [12]. Six comorbidities show decrease in the estimate of aHR indicating a potential overestimation of the strength of the association when only baseline information is used to predict mortality. Baseline survival models captured disease status at a single point in time. As a result, the interpretations of aHR for each chronic condition, collected at a single point in time, are attributed to the entire study period [15]. On the other hand, TCV models calculate a series of measures, breaking up the patient follow up time into smaller time windows. The start of each time window coincides with the onset of each additional disease and represents an increase in risk. TVC calculates a single weighted aHR for the entire study period using the series of aHRs from each time window. This allows TVC models to incorporate disease onset, changes in disease severity and exposure time, more accurately reflecting overall patient risk. The result of correctly classifying patients using TVC increases the average number of comorbidities for each patient while patient survival time remained the same. These findings have implications for existing and future studies using baseline assessment of comorbidity and highlight how disease misclassification could lead to errors in reporting.

Comparison between individual and summary measures shows that predictive models adjusting for all 17 individual Charlson comorbidities outperformed models using summary measures. Results from the literature are mixed. Austin et al. validated the performance and use of summary measures like the CCI and Elixhauser score as substitutes for individual comorbidities [14]. Sundararajan reported that using individual comorbidities performed better than the CCI, while Lieffers reported opposing results [16, 17]. Ghali suggested that summary measures calculated using study specific data had superior performance [18]. Acknowledging the variation in the literature, case mix adjustment using summary scores continues to be used in research for studies with small sample sizes.

Our comparisons and interpretations of aHRs between models took into consideration the natural history of the 17 Charlson comorbidities. Comorbidities with higher aHRs in TVC models compared to that in baseline models were considered to have higher risk of mortality in the short-term versus the entire study follow-up. Consistent with this interpretation, the aHRs and its 95% CI for hemiplegia or paraplegia (2.36, 2.29-2.43 in TIC and 2.89, 2.83-2.95 in TVC), were interpreted as a higher risk of death in short term following the onset of a disease or condition. Supporting this interpretation, Divanoglou reported that the highest mortality rate was within the first year of diagnosis or injury, with a 1-year mortality of 18.8% [19]. Conversely, a higher chronic obstructive pulmonary disease (COPD) aHR in the baseline model than in the TVC model could be interpreted as patients experiencing a low risk of death in the short term but with increasing risk in a longer timeframe. Shah and Kotloff [20] demonstrated that the natural history of COPD was slow to develop, with patients seeing minimal decline over short timeframes.

### Limitations

Our study focused on newly diagnosed adults with hypertension, a relatively healthy population requiring a long-term follow-up to observe mortality outcomes. Patients with newly diagnosed hypertension were identified over a 12-year period (April 1 1997 to March 31 2009), with a median follow-up time of 5.75 years, well short of median survival expected for hypertensive middle aged patients (median expected survival time of 20 + years). Future research using mortality as a study outcome should focus on populations with a higher risk of mortality, such as those with acute conditions or post intervention.

Use of administrative datasets may underestimate comorbidity burden for asymptomatic conditions (such as dyslipidemia or type 2 diabetes mellitus) in a relatively healthy, newly diagnosed hypertensive population. Without blood pressure measurement, asymptomatic hypertensive patients may not seek primary or acute care, and thus are not included in our study. The case definition we used has a high specificity (95 to 97%) but low sensitivity (66 to 72%), potentially under sampling low risk hypertensive patients [21].

Consistent with previous studies, we found that there was a lack of consistency and validated analytical procedures to compare improvements between TIC and TVC models [2].

## Conclusions

To the best of our knowledge this is the first large scale, population based study using administrative data investigating the onset of new comorbidities over time and its’ impact on predicting mortality risk in patients with a chronic disease. The resulting improvements in fit and performance of TVC predictive models were slight compared with method of using comorbidity assessed at baseline.

## Abbreviations

AHCIP: Alberta Health Care Insurance Plan
aHR: Sdjusted hazard ratios
AIC: Akaike information criterion
AMI: Acute myocardial infarction
CCI: Charlson comorbidity index
cHR: Crude hazard ratios
CI: Confidence intervals
COPD: Chronic obstructive pulmonary disease
DAD: Hospital discharge abstracts
ICD: International Classification of Disease
ICD-10-CA: International Classification of Disease 10^th^ revision Canadian Modification
ICD-9-CM: International Classification of Disease 9^th^ revision Clinical Modification
IQR: Inter-quartile range
log-HRs: Log hazard ratios
NR: Not reportable
PHN: Personal Health Number
Q1: Lowest quintile
Q5: Highest quintile
SD: Standard deviation
TIC: Time-invariant covariates
TVC: Time-varying covariates
